# Behavioural Difficulties in Children and Adolescents with Mental Disorders under Extreme Situations

**DOI:** 10.3390/jcm10214876

**Published:** 2021-10-22

**Authors:** Pablo Gonzalez-Domenech, José Luis Romero-Béjar, Luis Gutierrez-Rojas, Sara Jimenez-Fernandez, Francisco Diaz-Atienza

**Affiliations:** 1 Faculty of Medicine, University of Granada, Avenida Ilustración, 60, 18016 Granada, Spain; pgdomenech@ugr.es (P.G.-D.); gutierrezrojas@ugr.es (L.G.-R.); sarajimenezfer@hotmail.com (S.J.-F.); fdatienza@ugr.es (F.D.-A.); 2 Department of Statistics and Operational Research, University of Granada, Avda Fuentenueva, 18071 Granada, Spain

**Keywords:** COVID-19, confinement, emotional impact, mental disorders, children and adolescents

## Abstract

In 2020, the Governments of many countries maintained different levels of confinement of the population due to the pandemic that produced the COVID-19. There are few studies published on the psychological impact in the child and adolescent population diagnosed with mental disorders, especially during the home confinement stage. Explanatory models based on socio-demographic and clinical variables provide an approximation to level changes in different dimensions of behavioural difficulties. A categorical-response logistic ordinal regression model, based on a cross-sectional study with 139 children and adolescents diagnosed with mental disorders is performed for each dimension under analysis. Most of the socio-demographic and clinical explanatory variables considered (24 of 26) were significant at population level for at least one of the four dimensions of behavioural difficulties (15 response variables) under analysis. Odds-ratios were interpreted to identify risk or protective factors increasing or decreasing severity in the response variable. This analysis provides useful information, making it possible to more readily anticipate critical situations due to extreme events, such as a confinement, in this population.

## 1. Introduction

Public and private authorities have a growing research interest related to mental health, looking for, among others, the benefit of the community from an epidemiological point of view, as well as reducing the costs of, for example, health systems, etc. Particularly, mental health in children and adolescents has always had a special interest due to its impact in adult life. The child-adolescent population with mental disorders is especially vulnerable to extreme or extraordinary events, such as those experienced during the COVID-19 pandemic, particularly in relation to home confinement. Since the beginning of the pandemic, many countries implemented public health measures, such as home confinement, with the main goal of minimizing the spread of this virus [1,2]. For example, between 15 March and 20 June 2020, the Government of Spain maintained a state of alarm with different levels of confinement of the Spanish population due to the pandemic produced by the coronavirus disease 2019 (COVID-19).

In general, mental health involves anxiety, depression, suicide intentions, etc. There is wide and recent literature regarding the mental health consequences of these extreme public health measures [3,4,5]. Many studies show that young people are the most psychologically affected by an extreme situation, such as a confinement in this pandemic [6,7,8]. In addition, having psychiatric illness or psychological disorders is also associated to a worsening of mental health along periods of home confinement [9,10,11,12]. Despite the fact that children and adolescents suffer less severe physical symptoms due to COVID-19 [13], experts have warned of the emotional, behavioural, and social impact that the pandemic is producing in this sector of the population [14,15]. In China, the epicentre of this epidemic disease, the first publications about the psychological consequences of confinement in children appeared in March 2020 [16]. In many homes, parents had to combine work or teleworking with domestic chores and childcare during confinement, increasing levels of stress and family conflict, especially in children with some type of mental disorder and in single-parent families [17].

There are few studies published on the psychological impact of COVID-19 in the child and adolescent population diagnosed with mental disorders, especially during the home confinement stage. The most studied diagnosed are Attention Deficit Hyperactivity Disorder (ADHD) and Autism Spectrum Disorder (ASD) [18,19,20]. In general, all studies have found in their results an increase in emotional and behavioural alterations during confinement, but the risk factors involved have not been clearly identified and very few studies use a comparison with children without neurodevelopmental problems [18,21].

In this sense, this study has the purpose to identify factors increasing or decreasing severity in different dimensions of behavioural difficulties due to extreme situations, in patients diagnosed with internalizing and externalizing disorders according to DSM-5 classification [22].

## 2. Materials and Methods

### 2.1. Participants

The sample comprised 139 children and adolescents who regularly attend the Child and Adolescent Mental Health Unit (CHAMHU) in Granada. All these subjects were diagnosed as affected by internalizing (ASD, Emotional Disorders or Eating Disorders) or externalizing disorders (ADHD, Behavioural Disorders, or Intellectual Disability). It is important to mention that the main diagnosis of each participant in the case of comorbidities, for example autism + ADHD, was established based on giving PRIORITY to the SEVERITY criterion (which diagnosis causes the main disability) and TEMPORALITY (diagnosis is stable over time in the event that transitory diagnoses such as conduct disorders converge). The diagnosis of the participants was collected from the digitized clinical history of the CHAMHU of Granada and was included in the database using a labelling system that guaranteed anonymity. The mean age of the subjects was 11.4 years (SD = 3.9), 71.9% of them were male and 55.4% were diagnosed with internalizing disorders, while 44.6% with externalizing disorders.

### 2.2. The Procedure

A cross-sectional study comprising all the subjects was carried out. Data were obtained from an online survey by means of a standardized form model, Google Forms. The anonymous survey was sent via email (prior telephone authorization to parents) between 12 April and 25 May 2020 to a total of 328 parents of children and adolescents from the CHAMHU of Granada, who met the criteria to be accepted in this study. The inclusion criteria were an age range between 2 and 18 years and having an active follow-up (at least 12 previous months) in the CHAMHU of Granada. Participants who did not meet the two previous requirements were excluded. Those in whom, even meeting them, the reliability of the survey results could not be guaranteed (for example, parents with a mental or intellectual disability), or those in whom the context of the child was not that of a home confinement (i.e., institutionalized children) were also excluded. A total of 139 participants (the parents) responded to the survey. A random telephone survey was conducted among those who did not respond to the form, to determine the causes. Most of the parents reported having problems receiving e-mail, difficulties in completing the questionnaire, or lack of time.

### 2.3. Survey Design

The explanatory variables considered were selected according to the previous bibliography [23,24] and with the biopsychosocial model of psychiatry [25] that considers in the etiology of any mental disorder: biological factors such as sex, age or previous psychiatric diagnosis; psychological and interpersonal factors such as the level of family conflict and socio-cultural factors such as the type of housing or the maintenance of social relationships.

The form consists of five sections. Section 1 provides basic data information such as the sex, age, and date of birth of the subject. The next section reports family home conditions (coexistence with parents, number of children at family home, type of home, presence of pets, economic situation, and conflict existence before and during the confinement). Information related to family physical health is collected in Section 3. They are asked if any family member has passed away or has been sick due to COVID-19. Other illnesses in the family home are also reported. Section 4 refers to the emotional state of the child. This section provides information related to: if the subject was informed about the confinement reasons, concern degree of the child, and presence of behavioural disorders or changes. Finally, Section 5 reports on the management of the home during the confinement (sleeping and eating difficulties, fixed schedules in school homework, participates in household chores, physical activity, communication with other relatives or friends and teachers, other communications such as a psychologist or private teacher, use of information technology (ICT) and other difficulties).

The information reported by means of this survey is collected in statistical variables, measured in an ordinal scale (range 0–2) when it refers to responses variables. All the statistical variables, explanatory and response, are described in detail in Table 1 and Table 2.

### 2.4. Statistical Methods

One of the best statistical tools, because of its capacity for data analysis in clinical and epidemiological research, is logistic regression, hence its wide use [26]. Indeed, logistic regression provides information of which are the explanatory variables that can be considered as true risk factors because they involve level changes in the response variable. In this sense, it also provides information about the strength of these variables as risk factor by means of the interpretation of the odds-ratios (exponentials of the estimated parameters for each risk factor). If the response variable is a qualitative ordinal variable with more than two levels, then the ordinal logistic regression is adequate.

A cumulative ordinal logistic regression model with categorical ordinal response and qualitative explanatory variables was used [27,28]. Fifteen models were fitted for the response variables that report on the different difficulties that increased or decreased severity during the confinement. Each model was fitted in a stepwise way starting from a constant model, using forward selection to determine whether a variable enters, and backward selection to determine whether it exits, in each step. These models were used to determine which variables caused these subjects an increasing severity relative to the different categories of difficulties. The goodness-of-fit was compared using the Stukel ratio test, due to its robustness for the logistic regression model. The statistical significance of the parameters for the variables that can be considered as risk factors was evaluated using Wald’s test. The prognosis ratios for each level with respect to the adjacent levels were obtained, depending on the possible changes in the explanatory variables considered.

Statistical analyses were performed using the R Statistical Computing software 4.0.3 (https://www.r-project.org/, accessed on 19 October 2021).

### 2.5. Ethical Considerations

The study was carried out in accordance with the 1975 Declaration of Helsinki. The data were processed in accordance with the provisions of Act 3/2018, of 5 December, on the Protection of Personal Data and Guarantee of Digital Rights (LOPDGDD), and approved by the Ethics Committee of the Andalusian Health Service (TFG-IECS-2021).

## 3. Results

### 3.1. Description of the Sample

Difficulties due to confinement comprised of four categories: physiological disorders, social difficulties, behavioural disorders, and emotional disorders. These categories were described by means of 15 variables (response variables), whilst the considered explanatory variables (risk factors) were defined in 26 variables. The descriptive analysis of the response variables are shown in Table 1. The explanatory variables are described in Table 2.

### 3.2. Risk Factors for the Different Difficulties

The 15 estimated models related to physiological, behavioural, and emotional disorders, as well as social difficulties, include 24 of the 26 explanatory variables, at least for one of the response variables. The odds-ratios estimated for each explanatory variable in the cumulative ordinal logistic regression model, as well as their results of the Wald’s test, are shown in Table 3. This table has a double purpose: first (left to right), a physician can identify which are the relevant risk factors that involve level changes for each of the four categories of difficulties under analysis. Then (top to down) any of the explanatory variables are easily related with the different response variables that it influences. The results obtained by the Stukel test for each model, in Table 4, concluded that all the models, except the model related to the response variable Self-injury, produced a good fit at the population level.

According to the results of the Wald test, the variables *having a pet* (*p* = 0.041), *the mother suffers from a physical disease* (*p* = 0.045), *a brother or sister suffers from a physical disease* (*p* = 0.040), *global assessment during confinement* (*p* = 0.009), *behavioural problems during confinement* (*p* = 0.029), *maintains communication with other families* (*p* = 0.016), and *maintains communication with friends* (*p* = 0.044) are significant at the population level for at least one of the dimensions considered for difficulties related to physiological disorders. The only dimension of social difficulties is influenced by *having a pet* (*p* < 0.001) and *behavioural problems during confinement* (*p* = 0.002). Regarding behavioural disorders, the variables *age* (*p* = 0.006), *gender* (*p* = 0.006), *use Information and Communication Technology* (*p* = 0.008), *level of conflict before confinement* (*p* = 0.001), *level of conflict during confinement* (*p* = 0.020), *a close family member has died of COVID-19* (*p* = 0.011), *some family member is affected by COVID-19* (*p* = 0.006), *the mother suffers from a physical disease* (*p* = 0.037), *a brother or sister suffers from a physical disease* (*p* = 0.006), *behavioural problems during confinement* (*p* = 0.006), *maintains communication with other family* (*p* = 0.003), and *maintains other types of contacts* (*p* = 0.009) are significant for at least one of the dimensions of this category of difficulties. Finally, the variables *economic situation* (*p* = 0.012), *having children* (*p* = 0.026), *having a pet* (*p* = 0.011), *type of family home* (*p* = 0.007), *use Information and Communication Technology* (*p* < 0.001), *level of conflict before confinement* (*p* < 0.001), *level of conflict during confinement* (*p* < 0.001), *the mother suffers from a physical disease* (*p* = 0.023), *behavioural problems during confinement* (*p* = 0.003), *participates in household chores* (*p* = 0.001), *physical activity* (*p* = 0.004), *maintains communication with other families* (*p* = 0.011), *maintains communication with friends* (*p* = 0.022), and *maintains other types of contacts* (*p* = 0.002) are significant at the population level for at least one of the dimensions within the category related to emotional disorders.

The previous paragraph identifies risk factors for prognosis increasing or decreasing severity for the dimensions considered at each one of the four categories of difficulties due to confinement. Taking into account the double purpose of Table 3, it is also important to identify what dimension are each one of the explanatory variables relevant (top to down interpretation) in. In this sense, the variables *economic situation*, *having children, type of family home, participates in household chores,* and *physical activity* are only significant in relation to emotional disorders. *Age, gender*, *a close family member has died of COVID-19, some family member is affected by COVID-19,* and *a brother or sister suffers from a physical disease* are significant at the population level only for behavioural disorders. *Having a pet* is significant for physiological disorders, social difficulties and emotional disorders. *Use Information and Communication Technology, level of conflict before confinement, level of conflict during confinement*, and *maintains other types of contacts* are relevant for both, behavioural and emotional disorders. The variable *maintains communication with friends* is relevant for physiological and emotional disorders, whilst *global assessment during confinement* is only significant for physiological disorders. The variables *the mother suffers from a physical disease* and *maintains communication with other family* are significant regarding physiological, behavioural, and emotional disorders. *Behavioural problems during confinement* is the only variable that is significant for the four groups of difficulties. Finally, only the variables *coexistence in the family nucleus*, *the father suffers from a physical disease,* and *maintains school work schedule* are not relevant for inference according to this sample.

On the other hand, among all the significant explanatory variables, their odds ratio identify the presence of protection factors, i.e., they induce decreasing severity in the level of the response variable, as well as the presence of real risk factors since they produce an increasing severity for the response variable. Indeed, regarding this fact, for example: *age* induces an improvement in the levels of *aggressiveness (objects)* since for older children or adolescents the possibility of being at the same level or at a better level is double (OR = 2.15, *p* = 0.006) that of the youngest, whilst it induces a worsening for *self-injury* because the possibility of being at the same level or on a worse level is double (OR = 0.49, *p* = 0.007) for youngest child or adolescents; *gender* also induce an improvement for aggressiveness (objects) because women multiply by three (OR = 3.17, *p* = 0.006) the possibility of being at a better level for this response variable with respect to the men’s group; etc. Table 3 can be interpreted in this sense for each significant explanatory variable (*p* < 0.05); when OR > 1, it will be a protection factor (understood, this fact, as an improvement level or decreasing severity for the response variable under analysis), whilst for OR < 1, one interprets conversely as a real risk factor (understood, this fact, as a worsening level or increasing severity for the response variable under analysis) compared with respect to the last level of the response variable. In this sense, for instance *having a pet* is a risk factor for *Physiological Disorders* and *Social Difficulties* but a protection factor for *Emotional Disorders*; *economic situation* and *type of family home* are protection factors for *Emotional Disorders*; *use Information and Communication Technology* and *level of conflict before confinement* are protection factors for *Behavioural and Emotional Disorders* whilst *level of conflict during confinement* is a risk factor for these dimensions; *behavioural problems during confinement* is a risk factor for the four dimensions under analysis; *the mother suffers from a physical disease* is a risk factor for *Physiological and Emotional Disorders* but a protection factor for *Behavioural Disorders*; *a brother or sister suffers from a physical disease* is a protection factor for *Physiological and Behavioural Disorders*; *global assessment during confinement* is a risk factor for *Physiological Disorders*; *participates in household chores, physical activity, maintains communication with other family,* and *maintains other types of contacts* are protection factors for *Emotional Disorders*, *maintains communication with other family* is also a protection factor for *Physiological and Behavioural Disorders*, *maintains communication with friends* for *Physiological Disorders* but a protection factor for *Humor Changes* and *maintains other types of contacts* for *Behavioural Disorders.* Here, it is also noted that *a close family member has died of COVID-19* has a high strength as risk factor for self-injury since the possibility of worsening is 100-fold (OR = 0.01, *p* = 0.011) if a close family member has died of COVID-19, whilst *some family member is affected by COVID-19* is a protection factor for this response and the possibility of improvement is 20-fold (OR = 20.6, *p* = 0.006) if some family member is affected by COVID-19, and for *No Collaboration* the possibility of improvement is 6-fold (OR = 5.98, *p* = 0.003) in this case.

## 4. Discussion

The aim of this work was to identify factors increasing or decreasing in severity in different dimensions of behavioural difficulties due to extreme situations, in patients diagnosed with internalizing and externalizing disorders. With regard to this objective, 15 explanatory models based on socio-demographic and clinical variables provided an approximation to level changes in the dimensions of physiological disorders, social difficulties, and behavioural and emotional disorders. These models were used to identify risk or protective factors increasing or decreasing in severity in the response variable. The results obtained showed that some of these variables were significant for the prognosis of potential change to a higher or more severe level in each of the dimensions of behavioural difficulties under analysis. In this sense, *economic situation*, *having children*, *type of family home*, *participating in household chores* and *physical activity* are only significant in relation to emotional disorders. *Age, gender, a close family member has died by COVID-19, a close family member is affected by COVID-19* and *a brother or sister suffers from a physical disease* are significant at the population level only with regard to behavioural disorders. Epidemiological studies show that manifestations of mental distress in the form of behavioural alterations are more frequent in younger children and in boys [29]. Our study and others with similar characteristics [23] replicate these same findings in a stressful situation, such as home confinement due to a pandemic. The presence of a pet at home is significant for physiological disorders, social difficulties, and emotional disorders. The use of ICT, level of conflict before and during confinement, and if the patients maintains other type of contacts are relevant for both, behavioural and emotional disorders, according to other studies that highlight the relevance of communication between parents and children in the expression of psychopathology and discomfort [17,30]. The maintenance of communication with friends is relevant for physiological and emotional disorders, whilst the global assessment during confinement is only significant for physiological disorders. If the mother suffers from a physical disease and if the patients maintains communication with other family members are significant regarding physiological, behavioural, and emotional disorders. The presence of behavioural problems during confinement is the only variable that is significant for the four groups of difficulties. Finally, only the variables coexistence in the family nucleus, the father suffers from a physical disease and if the patients maintain the school work schedule are not relevant for inference according to this sample. It is possible that if our sample consisted only of patients with ADHD, these results would have been significant, as has happened with other works that have specifically studied this population [20,21].

From the results obtained, we propose a friendly-reading tabulation of the response dimensions and the socio-demographic and clinical variables, providing useful information for physicians. Indeed, it can be interpreted by rows (left to right) for quick identification of the dimensions affected by one of the socio-demographic or clinical characteristics. It can also be read by column (top to bottom) for quick identification of all the variables that influence a response characteristic or in the whole dimension of interest. Accordingly, we assessed the possible inclusion of these variables in a profile associated with child and adolescents with a high probability of experiencing increasing severity in behavioural difficulties under extreme situations. This conclusion was drawn on the basis of having found the influence, as risk or protective factor, of multiple socio-demographic and clinical variables for the four dimensions under analysis.

## 5. Conclusions

In view of the above considerations, we conclude than in order to establish a risk profile for behavioural difficulties among children and adolescents diagnosed as affected by internalizing or externalizing disorders, socio-demographic and clinical variables should be taken into account. This finding could be of great interest, and can be used as a basis for designing measures to prevent extreme situations among this population. Due to the considerable weight of these variables as risk or protective factors with respect to behavioural difficulties, we believe further research should be conducted, among populations of children and adolescents with these diagnoses and other children, in order to ratify the results in this paper. Furthermore, this would better facilitate the creation of risk profiles for each dimension of behavioural difficulties under extreme situations.

### 5.1. Study Limitations

One of the strengths of this work, conversely to other studies which are focused on isolated risk factors influencing a particular response variable [31,32], is that we face multiple risk or protection factors involving level changes for multiple responses variables associated to behavioural difficulties. However, there are inherent limitations. First, from a clinical viewpoint, the models estimated do not take into account other key variables for this purpose. For example, the presence of extreme adverse events such as physical and psychical aggressions [33,34,35]. The perception of insufficient information is a very common factor associated, in general, with poor mental health, but receiving negative news can involve high levels of anxiety and stress [36,37]. In this sense, it is also important to provide true information to these children and adolescents to avoid adverse behavioural responses.

### 5.2. Clinical Implications

Children and adolescents diagnosed as affected by internalizing or externalizing disorders are a risk group for behavioural difficulties under extreme situations because of the special nature of their disorder. This study identifies changes in these dimensions and determines which variables bear most weight, thus enabling us to predict and characterize changes in the severity of these behavioural difficulties. The analysis performed provides valuable information of risk and protective factors, facilitating the consideration of risk profiles for these difficulties, making it more readily identifiable and its evolution predictable.

## Figures and Tables

**Table 1:** Description of the response variables.

Difficulties	Response Variables	Level	% (*n*)
Physiological disorders	N = Sleep (*n* = 139)	(0) Worse	30.9 (43)
		(1) No changes	60.4 (84)
		(2) Better	8.7 (12)
	M = Nutrition (*n* = 139)	(0) Worse	31.7 (44)
		(1) No changes	58.9 (82)
		(2) Better	9.4 (13)
Social difficulties	G = Isolation (*n* = 139)	(0) Worse	33.1 (46)
		(1) No changes	36.0 (50)
		(2) Better	30.9 (43)
Behavioural disorders	L = Anger (*n* = 139)	(0) Worse	41.7 (58)
		(1) No changes	37.4 (52)
		(2) Better	20.9 (29)
	K = Aggressiveness (objects) (*n* = 139)	(0) Worse	37.4 (52)
		(1) No changes	29.5 (41)
		(2) Better	33.1 (46)
	J = Aggressiveness (people) (*n* = 139)	(0) Worse	32.4 (45)
		(1) No changes	30.2 (42)
		(2) Better	37.4 (52)
	I = Self injury (*n* = 139)	(0) Worse	29.5 (41)
		(1) No changes	30.9 (43)
		(2) Better	39.6 (55)
	F = No attention (*n* = 139)	(0) Worse	30.9 (43)
		(1) No changes	47.5 (66)
		(2) Better	21.6 (30)
	E = No collaboration (*n* = 139)	(0) Worse	28.1 (39)
		(1) No changes	43.9 (61)
		(2) Better	28.0 (39)
Emotional disorders	H = Irritability (*n* = 139)	(0) Worse	45.3 (63)
		(1) No changes	30.2 (42)
		(2) Better	24.5 (34)
	D = Apathy (*n* = 139)	(0) Worse	38.1 (53)
		(1) No changes	29.5 (41)
		(2) Better	32.4 (45)
	C = Sadness (*n* = 139)	(0) Worse	37.4 (52)
		(1) No changes	30.9 (43)
		(2) Better	31.7 (44)
	B = Easy cry (*n* = 139)	(0) Worse	40.3 (56)
		(1) No changes	43.9 (61)
		(2) Better	15.8 (22)
	A = Humor changes (*n* = 139)	(0) Worse	43.9 (61)
		(1) No changes	33.1 (46)
		(2) Better	23.0 (32)
	O = Fears (*n* = 139)	(0) Worse	38.9 (54)
		(1) No changes	34.5 (48)
		(2) Better	26.6 (37)

**Table 2:** Description of the qualitative regressors.

Socio-Demographic and Clinical Variables	Level	% (*n*)
DAG = Diagnostic (*n* = 139)	(1) Internalizing disorder	55.4 (77)
	(2) Externalizing disorder	44.6 (62)
AGE_C = Age (*n* = 139)	(1) Less than 6 years	10.8 (15)
	(2) 6 to 11 years	35.3 (49)
	(3) More than 11 years	53.9 (75)
SEX = Gender (*n* = 139)	(0) Male	71.9 (100)
	(1) Female	28.1 (39)
SIT = Economic Situation (*n* = 139)	(0) Bad	2.2 (3)
	(1) Regular	31.6 (44)
	(2) Good	66.2 (92)
CON = Coexistence in the family nucleus (*n* = 139)	(0) Both parents	74.8 (104)
	(1) Father	5.8 (8)
	(2) Mother	19.4 (27)
CHI = Having children (*n* = 139)	(1) One	32.4 (45)
	(2) Two	38.9 (54)
	(3) Three	18.7 (26)
	(4) >Three	10.0 (14)
PET = Having a pet (*n* = 139)	(0) No	46.8 (65)
	(1) Yes	53.2 (74)
VIV = Type of family home (*n* = 139)	(0) >90 m^2^	66.2 (92)
	(1) <90 m^2^	33.8 (47)
ICT = Use Information and Communication Technology (*n* = 139)	(1) Little	8.6 (12)
	(2) Moderate	47.5 (66)
	(3) Much	43.9 (61)
CFA = Level of conflict before confinement (*n* = 139)	(0) Low	53.2 (74)
	(1) Moderate	37.4 (52)
	(2) High	9.4 (13)
CFD = Level of conflict during confinement (*n* = 139)	(0) Low	41.0 (57)
	(1) Moderate	44.6 (62)
	(2) High	10.4 (20)
DF = A close family member has died of COVID-19 (*n* = 139)	(0) No	98.6 (137)
	(1) Yes	1.4 (2)
	(2) Not applicable	0 (0)
AF = Some family member is affected by COVID-19 (*n* = 139)	(0) No	91.4 (127)
	(1) Yes	8.6 (12)
	(2) Not applicable	0 (0)
EM = The mother suffers from a physical disease (*n* = 139)	(0) No	67.6 (94)
	(1) Yes	25.9 (36)
	(2) Not applicable	6.5 (9)
EP = The father suffers from a physical disease (*n* = 139)	(0) No	74.1 (103)
	(1) Yes	17.3 (24)
	(2) Not applicable	8.6 (12)
EH = A brother or sister suffers from a physical disease (*n* = 139)	(0) No	75.5 (105)
	(1) Yes	10.8 (15)
	(2) Not applicable	13.7 (19)
IH = The child has been informed of the reasons for the confinement (*n* = 139)	(0) No	3.6 (5)
	(1) Yes	96.4 (134)
EG = Global assessment during confinement (*n* = 139)	(0) Low concern	36.0 (50)
	(1) Moderate concern	43.2 (60)
	(2) High concern	20.8 (29)
PC = Behavioural problems during confinement (*n* = 139)	(0) No	92.8 (129)
	(1) Yes	7.2 (10)
I1 = Maintains school work schedule (*n* = 139)	(0) No	36.0 (50)
	(1) Yes	64.0 (89)
I2 = Participates in household chores (*n* = 139)	(0) No	23.8 (33)
	(1) Little	67.6 (94)
	(2) Yes	8.6 (12)
I3 = Physical activity (*n* = 139)	(0) No	52.52 (73)
	(1) Yes	47.48 (66)
I4 = Maintains communication with other family (*n* = 139)	(0) No	10.1 (14)
	(1) Yes	85.6 (119)
	(2) Not applicable	4.3 (6)
I5 = Maintains communication with friends (*n* = 139)	(0) No	30.2 (42)
	(1) Yes	64.8 (90)
	(2) Not applicable	5.0 (7)
I6 = Maintains communication with teachers or school counsellor (*n* = 139)	(0) No	23.0 (32)
	(1) YES	75.5 (105)
	(2) Not applicable	1.5 (2)
I7 = Maintains other types of contacts (*n* = 139)	(0) No	68.4 (95)
	(1) Yes	31.6 (44)

**Table 3:** Explanatory variables (OR and *p*-value). See Table 1 and Table 2 with codification of the explanatory and response variables.

	Physiological Disorders		Social Difficulties	Behavioural Disorders						Emotional Disorders					
	N	M	G	L	K	J	I	F	E	H	D	C	B	A	O
DAG				OR = 1.53 *p* = 0.057								OR = 1.49 *p* = 0.079		OR = 1.52 *p* = 0.071	
AGE_C	OR = 0.59 *p* = 0.058				OR = 2.15 *p* = 0.006		OR = 0.49 *p* = 0.007								
SEX					OR = 3.17 *p* = 0.006					OR = 1.84 *p* = 0.143				OR = 0.46 *p* = 0.062	
SIT		OR = 1.69 *p* = 0.144										OR = 2.46 *p* = 0.012			
CON							OR = 1.38 *p* = 0.132			OR = 1.48 *p* = 0.126		OR = 0.71 *p* = 0.155			
CHI															OR = 1.49 *p* = 0.026
PET	OR = 0.52 *p* = 0.076	OR = 0.47 *p* = 0.041	OR = 0.30 *p* < 0.001				OR = 1.93 *p* = 0.052						OR = 1.76 *p* = 0.108	OR = 2.46 *p* = 0.011	
VIV				OR = 0.58 *p* = 0.155	OR = 0.49 *p* = 0.053			OR = 0.50 *p* = 0.063		OR = 0.33 *p* = 0.007					
ICT				OR = 2.35 *p* = 0.008						OR = 2.11 *p* = 0.024			OR = 2.93 *p* < 0.001		
CFA				OR = 3.18 *p* = 0.001	OR = 3.09 *p* = 0.002	OR = 4.04 *p* < 0.001	OR = 1.69 *p* = 0.059	OR = 2.27 *p* = 0.017		OR = 4.00 *p* = 0.001	OR = 1.68 *p* = 0.043	OR = 2.69 *p* < 0.001	OR = 1.69 *p* = 0.048	OR = 2.20 *p* = 0.003	OR = 2.02 *p* = 0.006
CFD	OR = 1.67 *p* = 0.070			OR = 0.43 *p* = 0.020	OR = 0.31 *p* < 0.001	OR = 0.38 *p* = 0.004		OR = 0.32 *p* = 0.001		OR = 0.10 *p* < 0.001					
DF							OR = 0.01 *p* = 0.011								
AF							OR = 20.6 *p* = 0.006		OR = 5.98 *p* = 0.003			OR = 3.60 *p* = 0.053			
EM	OR = 0.54 *p* = 0.045	OR = 0.54 *p* = 0.065			OR = 1.99 *p* = 0.037			OR = 0.53 *p* = 0.051					OR = 0.45 *p* = 0.023		
EP					OR = 0.61 *p* = 0.102							OR = 1.57 *p* = 0.106	OR = 1.76 *p* = 0.069		OR = 0.61 *p* = 0.068
EH		OR = 1.77 *p* = 0.040				OR = 1.45 *p* = 0.124		OR = 2.09 *p* = 0.006	OR = 1.43 *p* = 0.128	OR = 1.67 *p* = 0.052	OR = 1.55 *p* = 0.066			OR = 1.57 *p* = 0.057	
EG	OR = 0.50 *p* = 0.009			OR = 0.64 *p* = 0.077	OR = 0.61 *p* = 0.053					OR = 0.64 *p* = 0.090					
PC	OR = 0.31 *p* = 0.134	OR = 0.20 *p* = 0.029	OR = 0.04 *p* = 0.002		OR = 0.03 *p* = 0.006	OR = 0.04 *p* = 0.005	OR = 0.18 *p* = 0.023	OR = 0.10 *p* = 0.054	OR = 0.11 *p* = 0.008			OR = 0.06 *p* = 0.018		OR = 0.03 *p* = 0.003	
I1				OR = 2.10 *p* = 0.065				OR = 1.78 *p* = 0.125		OR = 2.13 *p* = 0.069					
I2		OR = 1.72 *p* = 0.098		OR = 1.78 *p* = 0.110							OR = 2.67 *p* = 0.001	OR = 2.88 *p* = 0.002	OR = 1.93 *p* = 0.038	OR = 1.68 *p* = 0.111	
I3				OR = 1.88 *p* = 0.097						OR = 3.20 *p* = 0.004				OR = 2.55 *p* = 0.007	
I4		OR = 3.55 *p* = 0.016		OR = 2.60 *p* = 0.051	OR = 4.76 *p* = 0.003	OR = 3.14 *p* = 0.026		OR = 4.64 *p* = 0.003	OR = 3.06 *p* = 0.012	OR = 2.87 *p* = 0.053					OR = 3.08 *p* = 0.011
I5		OR = 0.47 *p* = 0.044				OR = 0.59 *p* = 0.139								OR = 2.19 *p* = 0.022	
I7				OR = 0.33 *p* = 0.009				OR = 0.34 *p* = 0.010		OR = 0.25 *p* = 0.002	OR = 0.50 *p* = 0.050	OR = 0.47 *p* = 0.054	OR = 0.41 *p* = 0.018		OR = 0.53 *p* = 0.072

RESPONSE VARIABLES: N = Sleep; M = Nutrition; G = Isolation; L = Anger; K = Aggressiveness (objects); J = Aggressiveness (people); I = Self injury; F = No attention; E = No collaboration; H = Irritability; D = Apathy; C = Sadness; B = Easy cry; A = Humor changes; O = Fears (*n* = 139). EXPLANATORY VARIABLES: DAG = Diagnostic; AGE_C = Age; SEX = Gender; SIT = Economic Situation; CON = Coexistence in the family nucleus; CHI = Having children; PET = Having a pet; VIV = Type of family home (n = 139); ICT = Use Information and Communication Technology; CFA = Level of conflict before confinement; CFD = Level of conflict during confinement; DF = A close family member has died of COVID-19; AF = Some family member is affected by COVID-19; EM = The mother suffers from a disease; EP = The father suffers from a disease; EH = A brother or sister suffers from a disease; EG = Global assessment during confinement; PC = Behavioural problems during confinement; I1 = Maintains school work schedule; I2 = Participates in household chores; I3 = Physical activity; I4 = Maintains communication with other family; I5 = Maintains communication with friends; I7 = Maintains other types of contacts.

**Table 4:** Goodness-fit-of test for each model.

Response Variables	Stukel Statistic	df	*p*-Value
N = Sleep (*n* = 139)	1.864	2	0.394
M = Nutrition (*n* = 139)	1.029	2	0.598
G = Isolation (*n* = 139)	2.707	2	0.258
L = Anger (*n* = 139)	1.241	2	0.538
K = Aggressiveness (objects) (*n* = 139)	0.660	2	0.719
J = Aggressiveness (people) (*n* = 139)	2.302	2	0.316
I = Self injury (*n* = 139)	15.754	2	<0.001
F = No attention (*n* = 139)	3438	2	0.179
E = No collaboration (*n* = 139)	2.192	2	0.334
H = Irritability (*n* = 139)	1.716	2	0.424
D = Apathy (*n* = 139)	3.998	2	0.136
C = Sadness (*n* = 139)	1.221	2	0.543
B = Easy cry (*n* = 139)	1.400	2	0.497
A = Humor changes (*n* = 139)	0.919	2	0.632
O = Fears (*n* = 139)	0.477	2	0.788

Note: *p* > 0.05 means good fit at the population level according to the Stukel test.
